# Regulatory framework for health information systems in Ethiopia: A qualitative document analysis

**DOI:** 10.1177/18333583251383109

**Published:** 2025-10-26

**Authors:** Befekadu Elfiyos Dekita, Mokholelana Margaret Ramukumba

**Affiliations:** 1 University of South Africa, Addis Ababa, Ethiopia; 2 University of South Africa, Pretoria, South Africa

**Keywords:** data usage, health information system, health management information system, HIV and AIDS, monitoring & evaluation, regulatory framework, health information management, evaluation study, document analysis, evaluation

## Abstract

**Background:** The regulatory and legal contexts of health information systems (HIS) play a crucial role in the generation and utilisation of health information. However, in many low- and middle-income countries, the regulatory framework for HIS remains underdeveloped, often hindering effective data management and system implementation. **Objective:** To evaluate how HIS policies, strategy and protocols guide the use of the health management information system (HMIS) for HIV and AIDS monitoring and evaluation (M&E) in Ethiopia. **Method:** An evaluation study using qualitative document analysis was adopted. Purposive criterion sampling was used to select government policy documents. Data extraction tool and management strategy were implemented. Atlas.Ti version 8 was used for content analysis. **Results:** Ten documents met the required criteria. The guidelines in the documents emphasised data quality assessment through data quality assurance techniques, data quality tracking of completeness and timeliness logbook and District Health Information System Version 2 (DHIS2) checks. Evidence from documents revealed broad guidance in terms of the implementation of M&E processes. Content showed clear definitions of information use at different levels and strategies to promote culture of data use. **Conclusion:** The study revealed that Ethiopia has an adequate regulatory framework for HIS to support the implementation of the HMIS in M&E of the HIV and AIDS program. However, the documents also acknowledge challenges with the implementation of these guidelines. **Implications for health information management practice:** This work highlights the importance of HIS and HMIS to the entire health system, and the vital role played by health information managers (HIMs) in managing the resources, people, protocols, and datasets essential for a functional information system, and for ensuring secure access to data for evidence-based decision-making.

## Introduction

Public health decision-making relies on the timely availability of high-quality data and proficiency in its utilisation. According to the World Health Organization (WHO) (2012), the regulatory and legal contexts of health information systems (HIS) play a crucial role in the generation and utilisation of health information. These contexts establish mechanisms to ensure the availability, exchange, quality and sharing of data. Paying specific attention to legal and regulatory considerations is imperative to guarantee that HIS produce data of high quality and usefulness. To ensure this, it is essential to establish standards that guide the collection, reporting and use of data, along with implementing routine checks to maintain data quality through standardised operating procedures. These operating procedures, specific to each country, describe the roles and responsibilities of data users and administrators (WHO, 2017a). Moreover, having written guidelines that specify methods and products with clear directions is crucial for effective data analysis. Transforming data into interoperable formats accelerate analysis. Mgbere et al. (2018) also emphasised the importance of guidelines for achieving consistency and efficiency in healthcare practices and noted that policymakers, health professionals and financiers, view guidelines as an essential tool for aligning clinical practices with government support.

However, digital healthcare data are often unstructured and lack standardisation, requiring significant pre-processing and normalisation, which poses challenges for integrating such data with other data sources (Sedlakova et al., 2023). Furthermore, the absence of standardised guidelines, tools and techniques to address these issues, which suggest that even well-resourced healthcare systems might be facing similar challenges. Internationally, while most eastern Mediterranean region countries have produced national HIS plans, some are inadequate in scope, particularly in terms of data collection, analyses and capacity-building (WHO, 2014). HIS in low- and middle-income countries (LMICs) are highly complex and influenced by pressures from donors, as well as political and administrative role players, which make accountability and good governance imperative (Koumamba et al., 2021). Yet, regulatory frameworks for HIS in LMICs are inadequate (Ojo, 2017). Koumamba et al. (2021) conducted a study across 12 African countries and while Malawi was found to have a comprehensive national strategy for HIS, the other countries relied on project-specific strategies. The WHO (2014) assessment also identified specific weak areas and provided valuable insights; for example, on the development of Mozambique’s new National HIS Strategy (Karuri et al., 2014). In response to identified shortcomings in Kenya’s HIS, the Ministry of Health took proactive measures to develop and integrate a comprehensive HIS strategy. Similarly, the Tanzanian Government embarked on strategic initiatives to tackle HIS challenges, as delineated in its Health Sector Strategic Plan, which aims to streamline health indicators, refine data collection and analysis methodologies and enhance overall system integration (Karuri et al., 2014).

A study conducted in north-western Ethiopia has drawn attention to significant challenges in accessing the health management information system (HMIS) data management guidelines, which in turn, impacted the utilisation of routine health information at the health centres level (Asemahagn, 2018). Bogale (2021) also highlighted the fragmented legislation within the Ethiopian HIS, particularly in relation to notifiable diseases, private sector data and vital statistics, fundamental principles of official statistics and confidentiality (Federal Ministry of Health [Ethiopia], 2012). In summary, the collective evidence underscores the need for improved regulatory frameworks and standardised strategies to enhance the effectiveness of HIS in LMICs.

### The current research

Because data use and decision-making rely heavily on the availability of guidelines and policies for the functioning of the health system (Dekita and Ramukumba, 2024), the goal of this study was to evaluate the importance of the regulatory framework for HIS in Ethiopia. Our evaluation aimed to gain insights into how policies, strategy and protocols guide the production of quality data and the use of information by data producers and users for HIV and AIDS monitoring and evaluation (M&E) in Ethiopia. To the best of our knowledge, no other recent studies have specifically examined legal and policy frameworks for HIS in Ethiopia to assess their adequacy for effectively evaluating their impact on the quality and usefulness of data for evaluating and monitoring HIV and AIDS in Ethiopia. The research question derived from this objective was how do policies, protocols and strategies guide the use of HMIS for HIV and AIDS M&E in Ethiopia?

## Method

### Study design

An evaluative case study approach using qualitative document analysis (QDA) was adopted (Morgan, 2012). This involved in-depth consideration of the nature of the case, physical setting and other institutional and political contextual factors (Ebneyamini and Moghadam, 2018). The case in question was HMIS implementation; the unit of analysis was the regulatory framework. Understanding the case required examining the content within the context of the country’s legislation.

The QDA offers a systematic methodological process for eliciting meanings from documentary evidence, serving as an overarching descriptor for a systematic, reflexive methodological process that encompasses various techniques such as content analysis, thematic analysis and discourse analysis (Wood et al., 2020). This use of QDA aligns methodologically with the constructivist worldview underpinning this study. Yin (2013) argued that document analysis yields valuable insights due to documents’ manageability, accessibility, cost-efficiency and stability over time. Particularly for this study, understanding the contextual factors surrounding the regulations guiding HMIS and HIS implementation necessitated a thorough examination of relevant documents. Importantly, documents serve as invaluable data sources in scenarios where events are no longer observable or participants’ recollection of details may be unreliable (Bowen, 2009). For this study, the researchers needed to understand the context in which the HMIS and HIS were implemented. Therefore, relying on document analysis was not only methodologically sound but also practical and efficient for capturing essential information crucial to the study objectives.

### Documents sample and sampling strategy

According to Gray et al. (2017), the study population should encompass all the compulsory elements that suit the main criteria for inclusion in the research. In our study, the document corpus included all documents that had pertinent information on the use of HMIS for HIV and AIDS M&E identified through key search terms and assistance from the Federal Ministry officials. Data sources included country-level developed HIS, HMIS/M&E documents that were currently in use. The Federal Ministry officials provided access to the data backup and base to search the documents. The search key words were HMIS, HIS, M&E, HIV and AIDS, Ethiopia, and they were used in conjunction with AND/OR.

Purposive criterion sampling was used to select information-rich documents to address the research question, guided by specific inclusion criteria (Creswell, 2014). Documents were included if they pertained to HIS, HMIS/M&E and the HIV and AIDS program. Additionally, selected documents were those directly providing guidance on data generation and management at the health facility level, specifically for the HIV and AIDS program. The timeframe for inclusion spanned the years 2008 to 2018. The rationale for this timeframe was to coincide with the HMIS implementation period in Ethiopia, with sampling conducted in 2018. Exclusion criteria included documents that were policy/framework/guidelines but unrelated to HIS, HMIS implementation and monitoring of the HIV and AIDS program at the health facility level and historical documents that had been amended or no longer in use. One Federal Ministry official with a doctorate in health informatics and governance provided support to the lead researcher as an independent analyst, to determine eligibility based on inclusion criteria. A point of consensus was made between the analysist and lead researcher. This meticulous selection process aimed to ensure that the chosen documents were relevant, current and directly aligned with the focus of the study. Three groups of documents were distinguished:

(a) Strategic documents that addressed regulatory issues related to the implementation plan and roadmap for successful implementation of the HMIS and HIV and AIDS program.
(b) Policy/framework documents directly related to the HMIS/M&E on the HIV and AIDS program.
(c) The guidelines/manual to guide implementation as per the required standards.

### Data collection approach

Data were collected according to the QDA assumptions. The researchers designed the data extraction tool based on a literature review and improved the tool through an iterative process based on the research question and field tests. Drawing from Wood et al.’s (2020) insights, the tool was strategically developed as a cost-effective means of structured data collection that ensured the collection of sufficient and pertinent information to address the research question.

All of the documents relevant to this study were found in grey literature databases, not conventional peer-review databases. This involved identification of relevant electronic sources in government and other websites, using the same keywords as those stated above. The search criteria included full-text published reports, policies, guidelines/manual and strategic/planning documents from 2008 to 2018; the entire process was documented for transparency, to be replicable and for possible reanalysing (Bengtsson, 2016).

**Figure 1.**
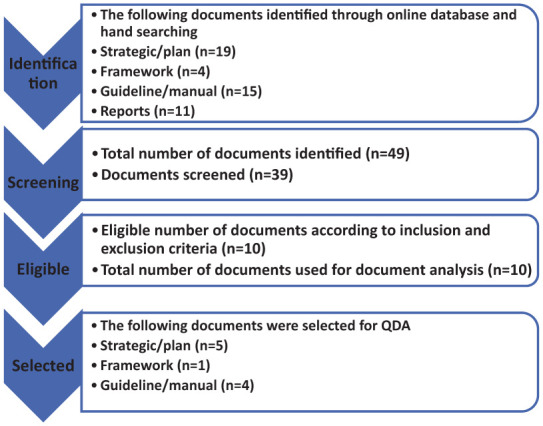
Flow diagram for the document search process.

The grey literature databases searched were Ethiopian Health Ministry Websites, WHO Ethiopian country Office, United States Agency for International Development (USAID) Ethiopia, Ethiopian National Archives and Library Agency; and 49 documents were collected from the online websites and archival system (see Figure 1). The documents were checked according to the inclusion and exclusion criteria (described under sampling strategy). A type of preliminary content analysis was conducted with the data extraction tool to identify information-rich documents (Wood et al., 2020). The independent analyst and the lead researcher compared documents selected for inclusion. Refinement was done until a point of consensus was reached. Quality assessment was not conducted as these were policy documents, not scientific papers. All selected documents were numbered/labelled alphabetically and numerically and then arranged for analysis.

### Data extraction

The data extraction tool was used to identify key components that aligned with the study objective and standardise the process across the documents. The extraction process involved selecting pertinent information from the chosen document, consolidating it on OneNote and subsequently exporting the compiled data to the extraction form, ready for thorough content analysis. This approach ensured that relevant and meaningful data were systematically gathered and prepared for further analysis, aligning with best practices in data extraction techniques. The process included identifying content related to the background and goals of the policy documents, key provisions or action points, roles and responsibilities of stakeholders. This included verbatim excerpts for accurate meaning, summaries of relevant sections, including page references to trace the origin of extracted data. The extracted data were stored in a spreadsheet for easy retrieval and comparison.

### Data analysis

The study utilised content analysis, described by Krippendorff (2004) as “a research technique for making replicable and valid inferences from texts (or other meaningful matter) to the contexts of their use.” Data were presented in words and themes, which made it possible to draw some interpretation of the results (Bengtsson, 2016). The study focused on the content of documents to identify the aspects related to HMIS regulatory issues, the context, to identify the rationale for regulatory issues, stakeholders and the implementation plan to ensure consistency in organising key findings. The researchers repeatedly read the documents to immerse themselves in the content, in order to find the content central ideas and meaning. To understand the content, diverse reasoning strategies were applied. These were inductive reasoning, analysis and synthesis. In the inductive approach, the organisation phase included open coding, creating categories and abstraction (Elo et al., 2014). Atlas.ti.version 8 software Lumivero, Denver/Colorado, United States was used for coding qualitatively, which improved stability and reliability when grouping data together in categories (Bengtsson, 2016). Independent coding was done by the analyst who was involved in the sampling stage for confirmatory process and to minimise researcher bias (Creswell, 2014). The researcher and the analyst repeatedly read the coded list to group and form categories and themes through consensus in each stage. The exploration stage involved a comparison across the themes to identify consistency, contradictions and trends. Coded sections were repeatedly read to align with the research question.

### Trustworthiness

This study used the Lincoln and Guba (1985) model criteria for trustworthiness: credibility, dependability, transferability and confirmability. To establish credibility and dependability, the researchers provided a transparent and detailed account of the entire process. This encompassed elucidating the rationale and methodology behind document selection, delineating the data collection procedure in terms of method and duration and explicating the approach to data analysis and presentation (as described above). Furthermore, the criterion of transferability was addressed by providing a contextual description of the study. The clear outline of the study’s context, including specific aspects on regulation of data generation, data flow and reporting, data quality, infrastructure and resource allocation and data use, facilitated an understanding of the conditions under which the study was conducted. This approach enhanced, the potential for the findings to be applicable and transferable to other relevant settings. For example, the researcher used a comprehensive description of data that were grouped according to themes and categories. This meticulous attention to the Lincoln and Guba (1985) model criteria ensured that the study met rigorous standards of trustworthiness in its design and execution.

### Ethical considerations

This study was approved by the Institutional Review Boards of the Departmental Higher Degrees Committee of the Department of Health Studies University of South Africa Ethical Clearance Committee for Research on Human Subjects (HSHDC/792/2017) and Addis Ababa City Administration Health Bureau (A/A/H/B/574/227). Also, a support letter was written by the University of South Africa Addis Ababa Regional Office to the Addis Ababa City Administration Health Bureau. Support letters from the above organisations were given to the health bureau.

## Results

### Description of number and types of documents analysed

Ten documents met the required criteria, labelled D1–D10 (see Box 1). Characteristics and descriptions of the studies selected for inclusion in this study are shown in Table 1. The documents were produced by the Ethiopian Federal Ministry of Health (FMOH) and the Federal HIV/AIDS Prevention and Control Office (FHAPCO). Currently, the country is more focused on developing strategic approaches, and 50% of the documents (*n* = 5) reviewed were strategic documents. Almost one third (*n* = 3) of the selected documents directly addressed the HIS and HMIS (Table 1 compares HIS and HMIS strategies). The other documents partly addressed both the HIS and HMIS. Two of the documents produced by the FHAPCO were more focused on describing the HIV and AIDS M&E Strategic Plan implementation.

**Box 1.** Description of the selected documents.

S. No.	Document name	Author name, date, document type, number of pages	Key content related to HIS, HMIS and M&E	Document label
1	HMIS/M&E	Federal Ministry of Health [Ethiopia], 2008, Strategic, 58 pages	Use of appropriate technology, information use and quality of information, HMIS training. Support decentralised, action-oriented, evidence-based decision making, resulting in: − use of evidence-based M&E by managers and health workers at all levels of the health system to plan, monitor and improve performance.	D1
2	Health data quality training module	Federal Ministry of Health [Ethiopia], June 2018 Manual, 73 pages	Completeness, timeliness of information and tracking, data legibility, data accessibility, precision, confidentiality, data integrity, data relevance, quality assurance and DQA technique. This guide targets all staff working in the health care system of Ethiopia.	D2
3	DHIS2 Implementation plan	Federal Ministry of Health [Ethiopia], July 2017, Plan, 39 pages	DHIS2 rollout objectives, switching from the existing eHMIS application, the DHIS2 customisation, DHIS2 pilot-testing. The target audiences are staff working at the HMIS unit.	D3
4	National HIV/AIDS M&E framework and cost plan 2015–2020	Federal Ministry of Health [Ethiopia] & Federal HIV/AIDS prevention and control office, July 2017, Framework, 71 pages	HIV/AIDS strategic plan, national HIV and AIDS M&E framework and objective, national M&E system. The target audiences are HMIS and program staff working in the HIV and AIDS program at the health facility and each hierarchy level of office.	D4
5	HIV/AIDS Strategic plan	Federal HIV/AIDS prevention and control office & Federal Ministry of Health [Ethiopia], December 2014, Strategic, 77 pages	The HIV investment case, health system strengthening, performance measurement and priority areas in the six years of implementation. The target audiences are staff working in the HIV and AIDS program at the health facility and each hierarchy level of office.	D5
6	HMIS Information use guide	Federal Ministry of Health [Ethiopia], May 2013 Guideline, 59 pages	Health information, the purpose of HMIS, indicator, data quality dimensions, LQAS, LQAS decision rule table, data discrepancy issues, actions to improve data. The target audiences are all health workers and other stakeholders.	D6
7	HMIS procedures manual: recording and reporting procedure	Federal Ministry of Health [Ethiopia], June 2018, Manual, 192 pages	Health system, health care system, HIS, components of HIS, HMIS. The target audiences are health care staff and managers at all levels of the health system.	D7
8	Information revolution road map	Federal Ministry of Health [Ethiopia], April 2016, Strategic, 62 pages	Information revolution, quality of data, reformed HMIS, use of ICTs, gaps in knowledge and skill, information use, shortages of HIT personnel, performance monitoring and improvement framework and electronic health (e-health). Target audiences are all level health facility managers and staff.	D8
9	Information use training module	Federal Ministry of Health [Ethiopia], June 2018, Manual, 124 pages	Information use and quality of information, data demand, data use, the culture of information use, information use at the facility level, determinants of information use, data analysis, interpretation, data presentation techniques and DHIS2 visualiser. Healthcare providers and managers at all levels of the health system are target audiences.	D9
10	National HIS road map	Federal Ministry of Health [Ethiopia], 2012, Strategic document/road map, 33 pages	HMIS and evidence-based decision, national HIS roadmap, routine HIS, HMN framework and standards, data management. All government and health development partners are target audiences.	D10

HIS: health information systems; HMIS: health management information systems; M&E: monitoring and evaluation; DQA: data quality assurance; DHIS2: District Health Information System Version 2; LQAS: lot quality assurance sampling; ICTs: information and communication technologies; HIT: health information technology; HMN: health metrics network.

**Table 1.** Ethiopian HIS and HMIS comparison on strategy.

S. No.	Areas of similarity and differences	Ethiopian HMIS (Document 1)	Ethiopian HIS roadmap (Document 10)	Comments
1	Strategic objectives	Five strategic issues have been identified as critical to strengthen and continuously improve health sector HMIS/M&E: • Capacity building. • Standardised and integrated data collection and reporting. • Linkage between information sources. • Information use; action-oriented performance monitoring. Appropriate technology.	There are five strategic objectives to achieve during and by the end of the 8-year period. These are: • To strengthen HIS governance, legislation, coordination and leadership. • To improve, strengthen and institutionalise HIS resources. • To improve health data coverage. • To improve health data management and quality. To strengthen and institutionalise information use for evidence-based planning, performance monitoring, feedback and action at all levels.	Ethiopian HMIS shows focus on harmonising three key issues, data generation and the use of information using appropriate technology. For HIS, the focus seems to be on governance and leadership and also institutionalising resources for proper data generation and information use.
2	Data flow	Establish data flow procedures that capture and transmit information in a timely fashion through an integrated reporting channel. Selected strategies: • Establish an integrated reporting channel that delivers information to the primary user when it is needed.	Make all routine HIS interoperable, including HMIS (HMIS, facility mapping, MRHIS, LMIS, infrastructure information systems, and IFMIS, LIS, RIS, mobile-health etc.).	With regard to data flow, HIS concentrates on regulation that will enable the systems to work together in an integrated format. Since the focus of the HMIS is on the use of information, data flow is supported through timelines and reporting channels.
3	Development of standards	• Establish standardised cascaded indicators for M&E at all levels: • Develop a standardised indicator set for the health sector and programs and disease list and case definitions for HMIS reporting. • Establish standardised client/patient recording procedures. • Standardised reporting instruments.	Ensure availability of appropriate infrastructure, standards, and tools for HIS. Develop a standardised data management system.	For the HMIS, emphasis was placed on standardising the indicators, recording procedures and reporting instruments. Whereas HIS concentrates on a standardised data management system through proper infrastructures and tools.
4	Capacity building	• Create the basic institutional structures and skilled staff to implement a well-functioning HMIS-M&E process. The first strategic issue addresses the need to institutionalise the HMIS-M&E responsibilities in the staffing structure and to establish pre-service and in-service HMIS-M&E training.	Strengthen the capacity of staff involved in HIS through in-service training.	Both the HMIS and HIS consider strengthening in-service training for capacity building of the staff. However, the HMIS also places emphasis on pre-service training and staffing structure.
5	Information use	• To strengthen and institutionalise information use for evidence-based planning, performance monitoring, feedback and action at all levels.	• Strengthen and ensure the functionality of performance monitoring teams at all levels. • Strengthen information dissemination mechanisms and use.	Both HIS and HMIS focus on performance monitoring and information use. However, HIS is viewed from the perspective of system functionality and mechanisms for information use, whereas the HMIS focuses on details surrounding planning, implementing, evaluation and feedback on information use.

HIS: health information systems; HMIS: health management information systems; M&E: monitoring and evaluation; MRHIS: medicine registration health information systems; LMIS: logistics management information system; IFMIS: integrated financial management information system; LIS: laboratory information systems; RIS: regulatory information system.

Three themes with related categories emerged from the data: Quality data generation and reporting, availability of resources and functionality of the HMIS/M&E system and data demand and use (Table 2).

**Table 2.** Themes and categories.

Theme	Category
Quality data generation and reporting	• Data generation and management. • Data quality.
Availability of resources and functionality of HMIS	• Key resources. • Design of HMIS.
Data demand and use	• Data collection and analysis. • Evidence-based decision-making.

HMIS: health management information system.

### Theme 1: Quality data generation and reporting

#### Data generation and management

Findings highlight the HMIS’s reliance on data gathered from diverse healthcare levels and multiple sources, as documented across various materials. These sources encompassed domains such as service delivery, financial transactions, disease surveillance, logistical operations and human resource management. Moreover, the HMIS procedures manual delineated specific data collection tools tailored for the HIV and AIDS program, focusing predominantly on the accurate recording of client information and meticulous record-keeping practices. The emphasis appeared to be on the following:

Data sources of the Ethiopian HIS at facility level Health centres (HCs), Hospitals and private health facilities: Routine HMIS report & surveillance report from Public Health Emergency Management (PHEM), facility-based researches (sic) and surveys. (D7)

Data Collection Routine information system data sources use various standard recording tools to capture data and these tools could be log sheets, registers, tally sheets. (D4)

Personal data are not disclosed inappropriately, and that data in hard copy and electronic form are treated with appropriate levels of security (kept in locked cabinets and in password-protected files). (D2)

The significance of data stood out as a fundamental aspect of effective data management within the examined documents. Users were acquainted with the intricacies of data analysis, comprehending its pivotal role and grasping its diverse applications. This understanding empowered stakeholders to succinctly summarise and compare data across various units or levels, thereby discerning emerging trends and identifying areas requiring attention. Documents highlighted the importance of information sharing through charts and tables. These should be visible and readily available at each health facility to facilitate use of information by stakeholders. Furthermore, a prominent focus of the documents was on defining the procedures and protocols for reporting specific indicators, both in periodic and annual reports. The following statements were extracted from the documents to show the emphasis:

Data analysis is the process of systematically applying different techniques to describe, summarise and compare data. It is the iterative process of examining data for patterns, trends, and comparisons . . . the program manager will compare the antiretroviral therapy target with the actual performance for the specific time period. (D9)

The data from health facility levels is regularly sent to next reporting system. Overall, eighty-seven percent (sic) of health facilities report data through the government reporting system. (D10)

#### Data quality

The guidelines within the document notably focused on data quality assessment, employing various techniques such as data quality assurance (DQA), District Health Information System Version 2 (DHIS2) checks, and the tracking of completeness and timeliness through a logbook. Lot quality assurance sampling was highlighted due to its importance in assessing routine HIS performance, particularly in terms of producing quality data and facilitating evidence-based decision-making:

Quality of data is a key factor in generating reliable health information that enables monitoring progress and making decisions for continuous improvement. . . . The overall DQA and the tools that are commonly used are LQAS, performance monitoring team, and ISS, with specific focus on data use. (D8)

DQA: a systematic M&E of data to uncover inconsistencies in the data and data management system . . . DQA techniques: Data Quality Desk review. The DHIS2 will give the users a message that the value entered is not the correct format . . . All health facilities have timeliness and completeness tracking logbook. (D2)

### Theme 2: Availability of resources and functionality of the HMIS

#### Key resources

Documents reviewed indicated that adequate staffing was important to the success of the utilisation of the HMIS in HIV and AIDS program. The content of some documents clearly showed the need for training or capacity building for technical staff working with the HMIS/M&E, whether in-service or pre-service training:

Adequate skilled human resources should be ensured at all levels of the M&E system in order to complete all tasks . . . (D4)

Training in both HMIS technical tasks and in action-oriented monitoring is needed. For M&E, in-service training is needed . . . This training should focus on problem-solving as well as interpretation of information. (D1)

Enhance HIS staff career development opportunities through improving the capacity of health professionals, M&E personnel and health managers . . . strengthen human resources for health capacity to effectively use ICTs [information and communication technologies]. (D8)

The emphasis seemed to be in recognising the pivotal role of manuals or guidelines, serving as essential tools to direct the health professionals in data generation, data collection, analysis and display and reporting tasks. Data mined from documents indicated that national indicators should guide program M&E. Particularly in the HIV and AIDS program, key performance indicators play a crucial role in evaluating the alignment of the plan with actual achievements:

Manual is primarily intended for use by health workers at all levels of the health system who are involved in managing health related data. (D7)

Indicators measure the value of the change of a single aspect of a program or project . . . key performance indicator for HIV/AIDS program are clients receiving voluntary counselling and testing services, prevention of mother to child transmission treatment completion rate and patient currently on antiretroviral therapy. (D6)

ICTs and other related resources were recognised as critical for the efficiency and effectiveness of healthcare delivery. The content of documents supported the utilisation of DHIS2 to enable data generation activities such as data entry/capture, analysis and validation/checking of data:

The health sector must invest significant resources to leverage these ICT investments . . . applications including telemedicine, tele-education, mobile health, electronic HMIS (eHMIS), electronic medical records (EMR), geographic information systems (GIS) and human resource information systems (HRIS). (D8)

Ensure all regional HIV/AIDS bureaus and woreda level^1^ will have sufficient computers and internet access . . . shortage of supplies and equipment for M&E functions (tools, computers, printers, photocopiers) and poor infrastructure (electricity, telephone services and internet access). (D4) (woredas are 3rd level of administrative divisions of Ethiopia).

DHIS2 has several features that support initiatives for improving data quality; validation during data entry to make sure data is captured in the right format. (D2)

#### Design of the HMIS

The documents highlighted the essential role of a fully functioning health system, with HIS recognised as one of its foundational building blocks. Specific content presents the HMIS mission, vision, goal and strategic plan. The significance of the HMIS in M&E is acknowledged, underscoring its invaluable contribution to informed decision-making and overall health system performance:

Health system is the sum total of all the organisations, institutions, resources and all activities . . . health system has six components/building blocks. These include: Service delivery, health workforce, health financing, health information system, medical products and governance & leadership. (D7)

The HMIS policy includes the people, procedures, datasets, hardware and software that are essential to coordinate a functional information system and ensure that facilities use the information generated in decision-making. (D1)

The content revealed that a data management plan was critical in M&E. The need to strengthen M&E was acknowledged. The relationship between strategic plans in response to HIV and AIDS using information was made explicit in the documents. Plans needed to be formulated and communicated to various stakeholders:

Realisation of a simple, coordinated, unified and effective results-based national M&E system for data management, dissemination and utilisation of strategic information for the HIV/AIDS response . . . an efficient and effective M&E system will enable informed decisions to improve performance. (D4)

The primary purpose of communication is to gain support and approval for the annual HIS plan and budget proposal. (D1)

### Theme 3: Data demand and use

#### Data collection and analysis

Various stakeholders needed different types of information in varying detail to support decision-making. The plan needed to reflect a multi-sectorial response to HIV using the performance of indicators as a basis for action:

Data demand is related to the value stakeholders attach to data. We say that data demand exists if: specific questions are raised and data are considered to answer them . . . evidence-based decision-making practice is influenced by demand for health information. (D9)

The preparation of the next five years sector strategic plan, this is a good opportunity to incorporate the multi-sectorial HIV response performance indicators to the information management systems. (D5)

#### Evidence-based decision-making

Content showed clear definitions of information use at different levels and processes. The need for evidence-based decision-making and use of decision tracking matrix were emphasised:

Use of information for decision making has been part of the M&E system of the HIV/AIDS response since inception . . . available information needs to be disseminated in a timely manner and used for strategic decision making at all levels of the health system. (D4)

Performance monitoring well-designed and documented data sources, M&E structures, availability of guidelines, finance, and skilled staff are all key resources for an effective performance monitoring system . . . performance improvement framework, teams should monitor performance compared to planned targets using different decision support tools. (D8)

Performance monitoring team (PMT) must be established at all levels of the health system, . . . heads or delegate heads of the institution are the chair persons (sic) of the performance monitoring teams at all levels; planning and M&E unit head or HMIS focal person will be a secretary . . . performance monitoring team meeting should be planned to be conducted regularly. (D9)

## Discussion

The study documents have emphasised the crucial role of political support in fostering a fully operational HIS, acknowledging HIS as a strategic foundation for a comprehensive health system (WHO, 2017b). Countries have an obligation to ensure that their national HIS is robust, secure and compliant with required standards. It is evident that the Ethiopian HIS framework serves as a foundational structure to facilitate the functioning of the HMIS and reinforces overall health system performance. The standards and guidelines governing the HIS reflect the roles and responsibilities of the different government sectors and donors for a multi-sectoral approach to ensure the efficacy of the HMIS. Within this framework, the HMIS policy encompasses hardware, software, procedures, datasets and human resources, all essential components for coordinating a robust information system and facilitating informed decision making at all levels of the healthcare delivery chain. Furthermore, these documents stress the reliance of HMIS on data collected from diverse sources, with specific recognition given to data collection tools tailored for the HIV and AIDS program. Notably, the documents emphasised the necessity for data features to be relevant, confidential, simple and easily accessible. Additionally, there was strong support for leveraging electronic platforms like DHIS2 to streamline data processing activities and enhance data quality.

Encouraging the local use of routine data recorded through HMIS tools at the service provision level demonstrates a commitment to fostering an information culture and strengthening knowledge management capacities. This concerted effort aims to empower stakeholders at all levels to utilise information effectively for informed decision-making and action, thereby driving improvements in program performance and health outcomes. The key issue with tools appears related to the hybrid version of manual and electronic reporting systems. The documents highlight the government’s plan to introduce the information revolution into health facilities through ICT. Manual and traditional approaches to data management at health facilities can often make data management tasks problematic (Kebede et al., 2020). In many LMICs, the HMIS regulations are also fragmented across agencies or ministries (Kumar et al., 2017). In Namibia, the HIS policy development has been very slow, with a resulting detrimental impact on data management processes (Kapepo and Yashik, 2018). Conversely, South Africa has strengthened its health policy development, although implementation weakened by inadequate leadership and limited resources (Lane et al., 2020).

Health systems require support in the analysis and interpretation and use of information at the district and facility levels (Nicol et al., 2017). The HMIS goal, as specified in the policy documents, is to support evidence-based decision-making in the health sector. It is evident that there is a framework that guides tasks involved in data analysis as well as the competencies required. The importance of information sharing and using charts, graphs and tables for prompt information use is adequately articulated.

Evidence from the documents revealed the crucial role of annual reports in providing insights into the specific health needs of the country, compiled from data at the facility level. Consequently, there is an imperative for these reports to embody qualities of accuracy, timeliness and completeness, all of which are integral dimensions of data quality. Furthermore, the guidelines within the documents underscore the significance of data quality assessment, emphasising the use of DQA techniques, standardised report formats and specific reporting periods (Cheburet and Odhiambo-Otieno, 2016). The process includes tracking data quality by using completeness and timeliness logbooks, as well as conducting checks through the DHIS2 system. Samal and Dehury (2016) argued that the benchmarks for quality within the HMIS framework are characterised by the timely, accurate and error-free reporting and uploading of data. This highlights the commitment to maintaining high standards of data quality within the HMIS, essential for informed decision-making and effective health system management.

DHIS2, indicators, ICT and other related resources are discussed here as part of the technical factors. ICT and other related resources are recognised as critical for efficient and effective healthcare delivery. The regulatory framework supports the utilisation of DHIS2. Documents revealed about acquiring sufficient tools and infrastructure to support the generation of quality and useful data. The WHO (2021) recommends the adequate provision of information and communication technologies for health to promote equitable, affordable and universal access. In Ethiopia, the HMIS indicators provide a portrait of the current health system and function (Ministry of Health-Federal Democratic Republic of Ethiopia, 2013). The importance of indicators to monitor the performance of the program, to show whether it is achieving its intended objectives or not, is acknowledged. The key performance indicators in the HIV and AIDS program are clearly described. Diana et al. (2017) articulates similar sentiments that the selection of indicators should be based on the importance of data collection choices. Literature also emphasises a need to harmonise indicators (Gloyd et al., 2016). As previously highlighted, the Ministry of Health formulated comprehensive procedures, guidelines and protocols for the HMIS, encompassing data management processes, data quality and information utilisation. An examination of documents through content analysis further underscores the pivotal role of adequate staffing in ensuring the success of HMIS/M&E for the HIV program, emphasising the importance of human resources in this domain.

There was a notable emphasis on capacity development within the documents, although challenges regarding shortages and turnover of health information management personnel were also acknowledged. Consistent with these observations, the study’s findings also indicated that scarcity of qualified personnel, particularly nurses and data entry operators responsible for data inputs, was a primary contributor to poor data quality (Tripathi et al., 2018). Consequently, the documents suggested a necessity for targeted resource mobilisation through staffing allocation and recruitment, accompanied by adequate budgeting to address these staffing challenges. This strategic approach aims to strengthen the human resources component, ensuring a proficient and sustained workforce capable of ensuring data quality standards and facilitating effective health program M&E. Developing countries experience a myriad of challenges with the implementation of HIS. There are various and often complex contributory factors involved, including, for example, lack of resources, which hamper efforts for successful use (Adaletey et al., 2014). Few LMICs have robust legal frameworks to address the protection of personal health information. A common issue for many is the absence of national standards for data exchange, which leads to challenges in integrating data from various sources (Jayathissa and Hewapathirana, 2023). However, Rwanda has developed a robust HMIS framework supported by the government digital strategy, based on a recent assessment of data quality using WHO guidelines for HMIS data verification (Nshimyiryo et al., 2020).

While high-income countries (HICs) have made substantial progress in developing national HIS, they can nonetheless learn valuable lessons from the challenges faced by LMICs in managing HMIS. The COVID-19 pandemic highlighted the significance of global data exchange frameworks and while several health innovations were researched and developed in LMICs during this period, they were rarely published. However, as highlighted in a scoping review by Ishimwe et al. (2023), HICs drew inspiration from health innovations researched and developed in LMICs and adapted and adopted these for their own use. However, to maximise the benefits of innovation transfer and exchange would require HICs to prioritise the development of cohesive, interoperable systems and proactively implement standardised data sharing protocols.

The contents of the documents showed that data demand was driven by the formulation of specific questions and there was an understanding that data should be available to answer these questions. Three documents outlined and described the HIV/M&E Plan, detailing costs and related activities. This plan presented a multi-sectorial response to HIV using the performance of indicators as a basis for action and the important relationship between information use and decision-making was well reiterated. The use of a decision-tracking matrix was emphasised, with plans for this to form the basis of decision-making for performance improvement. Resources required for performance monitoring were also highlighted, with emphasis placed on the availability of a monitoring plan that demonstrates the extent to which targets are achieved and the different tools that could be used to achieve them. Thus, the importance of the HMIS data and external monitoring systems were acknowledged and performance review meetings deemed critical approaches to safeguarding data quality and information use. As the literature shows, these findings are not exclusive to Ethiopia. Lippeveld and Hagan (2017) highlighted the challenges of creating a culture of data use essentially related to the information systems, the organisational and behavioural capacity building efforts and the economic issues in many LMICs. The MEASURE Evaluation (2014) also showed that for HIV M&E, a planned data demand is necessary for decision-making and the use of a decision tracking matrix.

### Limitations

This study focused on one country only. Inclusion of other LMICs would have provided a more meaningful evaluation of the HMIS regulation. The document review included only documents that were currently in use; historical documents were excluded. However, the broad range of documents selected provided adequate understanding of the context and content. There was a potential bias in the generalisability of the findings to other LMICs.

## Conclusion

The study concluded that Ethiopia has adequate HIS and HMIS policy infrastructure to sufficiently support quality data generation and proper data flow within the health system. The content and structure of these policy documents provide a framework and support the HIV and AIDS program. However, the documents also highlight challenges with reporting. Low quality of data was mentioned as leading to poor decisions. Information must be shared through graphs, charts and tables for prompt information use to succeed. The study results also acknowledged the critical nature of the regulatory framework to guide the development of internal structures for all data management processes. Policymakers must develop mechanisms for a periodical review of the policies and guidelines, especially in the light of constant changes in information needs. The need to strengthen M&E is acknowledged. Plans should form the basis for decision-making to improve performance, and the critical role of guidelines and protocols in the HMIS use needs to be strongly emphasised.

## Notes

1. “woredas” are third level of administrative divisions of Ethiopia.
